# Decylprodigiosin: a new member of the prodigiosin family isolated from a seaweed-associated *Streptomyces*

**DOI:** 10.3389/fphar.2024.1347485

**Published:** 2024-03-21

**Authors:** Mariana Girão, Sara Freitas, Teresa P. Martins, Ralph Urbatzka, Maria F. Carvalho, Pedro N. Leão

**Affiliations:** ^1^ Interdisciplinary Centre of Marine and Environmental Research (CIIMAR), University of Porto, Matosinhos, Portugal; ^2^ School of Medicine and Biomedical Sciences Abel Salazar (ICBAS), University of Porto, Porto, Portugal

**Keywords:** decylprodigiosin, *Streptomyces*, seaweed, bioactivity, actinomycete

## Abstract

Bioprospecting actinobacterial secondary metabolism from untapped marine sources may lead to the discovery of biotechnologically-relevant compounds. While studying the diversity and bioactive potential of Actinomycetota associated with *Codium tomentosum,* a green seaweed collected in the northern Portuguese cost, strain CT-F61, identified as *Streptomyces violaceoruber*, was isolated. Its extracts displayed a strong anticancer activity on breast carcinoma T-47D and colorectal carcinoma HCT116 cells, being effective as well against a panel of human and fish pathogenic bacteria. Following a bioactivity-guided isolation pipeline, a new analogue of the red-pigmented family of the antibiotics prodigiosins, decylprodigiosin (1), was identified and chemically characterized. Despite this family of natural products being well-known for a long time, we report a new analogue and the first evidence for prodigiosins being produced by a seaweed-associated actinomycete.

## Introduction

Nature is a wealthy reservoir of biotechnologically-relevant molecules, some of them even labelled as prodigious (i.e., something marvellous), as the family of the blood-red pigmented bacterial alkaloids, prodigiosins. This group of compounds harbours a diverse set of heterocyclic natural products (NPs), with historical evidence dating back to the beginning of the 19th century (Bennett and Bentley, 2000). Surveying the broad spectrum of properties that prodigiosin and prodigiosin-related molecules encode, from antimicrobial, antimalarial, anticancer, algicidal, antiparasitic to antiviral and immunosuppressive (Castro, 1967; Montaner and Prez-Toms, 2003; Lapenda et al., 2015; Zhang et al., 2016; Zhou et al., 2016; Ehrenkaufer et al., 2020), it becomes clear why they have attracted the attention of NPs research programs for so many years. Apart from their value in medical industry, they are also used in food, cosmetics and dye markets as an eco-friendlier and cost effective alternative to synthetic pigments (Paul et al., 2020). Prodigiosins have been reported to be produced by a wide range of Gram-negative and positive bacteria. Examples include members of the Pseudomonadota phylum as *Alteromonas rubra*, *Achromobacter denitrificans*, *Beneckea gazogenes*, *Hahella chejuensis*, *Janthinobacterium lividum*, *Rugamonas rubra*, *Zooshikela rubidus*, (Harwood, 1978; Gerber and Gauthier, 1979; Austin and Moss, 1986; Schloss et al., 2010; Lee et al., 2011; Pradeep et al., 2014)*, Pseudoalteromonas* (Kawauchi et al., 1997; Klein et al., 2017), *Serratia* (Williams, 1973; Berg, 2000; Miao et al., 2013; Su et al., 2016; Halder et al., 2020) and *Vibrio* (Allen et al., 1983; Vitale et al., 2020). Members of the phylum Actinomycetota, considered the most prolific bacterial source of drug-lead chemicals (Bahrami et al., 2022), have also been reported to synthetize prodigiosins. From several species of *Streptomyces* (*S. longisporuber*, *Streptomyces griseoviridis* and *S. coelicolor*), *Actinomadura* (*Actinomadura madurae* and *A. pelletieri*), and from *Streptoverticillium rubrireticuli*, undecylprodigiosin, butylcycloheptylprodigiosine, metacycloprodigiosin, nonylprodigiosin, prodigiosin 25-C and prodigiosins R1, R2 and R3 were described, highlighting actinobacterial metabolism richness in the production of these tripyrrole red pigments (Harashima et al., 1967; Gerber, 1969; Gerber, 1975; TSAO et al., 1985; Kawasaki et al., 2008; Kimata et al., 2018; Islan et al., 2022; Kimata et al., 2023). The widespread occurrence of prodigiosins in phylogenetically distant microbes suggests that these molecules may play a significant, albeit uncertain and yet to be fully defined, physiological role. In recent years, marine-sourced Actinomycetota have proved their value as source of relevant chemistry (Girão et al., 2022). Some known prodigiosins and related molecules, exhibiting a wide range of bioactive properties, have been found to be produced by *Streptomyces* species living in association with sponges (El-Bondkly et al., 2012; Abdelfattah et al., 2019) and inhabiting deep-sea sediments (Song et al., 2015). Also from a marine *Streptomyces*, two novel spiroaminals, marineosins A and B, obtained from unknown modifications of prodigiosin-like pigment pathways and exhibiting significant anticancer activity, have been discovered (Boonlarppradab et al., 2008). *Streptomyces* can be found in many places in the marine ecosystem, including in association with seaweeds (Girão et al., 2019), but little is known regarding bioactive NPs biosynthesis as part of such associations. As prodigiosins display algicidal properties, they are able to inhibit and control the growth of various microalgae and cyanobacteria (Zhang et al., 2016; Yang et al., 2017; Zhang et al., 2017; Zhang et al., 2020), by disrupting cell membranes, interfering with photosynthesis, and/or inducting oxidative stress, having been considered good candidates for algal bloom management and aquatic ecosystem restoration. In this work, by exploring the secondary metabolism of the seaweed-associated *Streptomyces violaceoruber* CT-F61, isolated from the tissues of *Codium tomentosum*, a new 10-carbon alkyl chain prodigiosin was discovered and chemically characterized. To our knowledge, the presence of bacteria producing prodigiosins living in association with macroalgae has hitherto not been described. We briefly discuss the potential ecological significance of this finding.

## Materials and methods

### Sampling and bacterial isolation

One specimen of *Codium tomentosum* was collected in the intertidal rocky northern Portuguese shore (41.309298°; −8.742228°). The macroalga was transported to the laboratory under refrigeration and processed on the same day for Actinomycetota isolation. The seaweed was washed with sterile sea water and macerated using a sterile mortar. To increase the success of actinobacterial isolation, the macerated tissues were incubated in a water bath at 58 °C for 15 min, to limit the incidence of non-spore forming microorganisms and boost the development of slow growing strains. From a tissue sample inoculated in Actinomycete Isolation Agar (AIA)—4 g C_3_H_5_NaO_2_, 0.5 g K_2_HPO_4_, 0.2 g Na_2_CO_3_, 0.2 g FeSO_4_, 0.2 g MgSO_4_ and 0.1 g L-arginine per litre of a 3:2 mixture of deionized water/seawater, supplemented with cycloheximide and nalidixic acid (50 mg L^−1^; Sigma-Aldrich, MO, United States)—strain CT-F61 was isolated as an axenic culture.

### Taxonomic and phylogenetic analysis of *Streptomyces* violaceoruber CT-F61

Strain CT-F61 was taxonomically identified through 16S rRNA gene sequencing. Biomass of the strain was obtained from a 2-week old culture in AIA liquid medium, and its genomic DNA was extracted using the E. Z.N.A. Bacterial DNA Kit (Omega Bio-Tek, GA, United States). The 16S rRNA gene was amplified by PCR (universal primers 27F and 1492R (Weisburg et al., 1991)) and sequenced as described by Girão *et* al., 2019 (Girão et al., 2019). The obtained sequences were analysed using Geneious Prime 2023.1 software (Biomatters, Auckland, New Zealand). The taxonomic identification of CT-F61 was established by comparison of its 16S rRNA gene consensus sequence with type strains deposited in the EzBioCloud database (Yoon et al., 2017). To infer the evolutionary relationship between CT-F61 and its closest related species, a phylogenetic tree was built. The 15 consensus sequences closest to CT-F61 (according to ExTaxon database) were selected and aligned, together with CT-F61 16S rRNA gene sequence and *B. subtilis* NCIB 3610^T^ as an outgroup, using the MUSCLE tool from the Geneious software package. An alignment of 1366 bp was used to construct the phylogenetic tree, applying the Maximum Likelihood method with 1000 bootstraps based on the Tamura-Nei model. Evolutionary analyses were conducted in MEGA X (Kumar et al., 2018).

### Bioactivity screenings

Strain CT-F61 was cultured in AIA medium, and its organic extract obtained, following the methodology previously described in Girão *et* al. (Girão et al., 2019). This crude extract was tested against a panel of human and fish pathogenic microbial strains (Table 1), using the agar-based disc diffusion method. The assay was performed with the turbidity of each test organism adjusted within the 0.5 McFarland standard (OD_625_ = 0.13) in the corresponding culture media. The suspensions were used to inoculate agar plates by evenly streaking each with a swab dipped in the suspension. In each blank disk (6 mm in diameter, Whatman, UK), placed on the surface of the inoculated plates, 15 µL of the crude extract (1 mg mL^-1^, dissolved in dimethyl sulfoxide DMSO ≥99.9%; Sigma-Aldrich, MO, United States) were loaded and the plates incubated accordingly to the reference organism requirements (Table 1). Antimicrobial activity was determined by measuring the diameter of the inhibition halo formed around the disc. Positive controls consisted in Enrofloxacin (1 mg mL^-1^) for all bacterial strains and Nystatin (1 mg mL^-1^) for the yeast *Candida albicans*. DMSO was used as negative control for all the microorganisms. The crude extract was tested in triplicate, in two independent experiments. Results are presented as the average diameter of the inhibition halos measured (mm) in each experiment. The cytotoxic activity of CT-F61 crude extract was tested at a concentration of 15 μg mL^-1^ in monolayer cell cultures of two cancer and one non-cancer cell lines—T-47D cells (breast ductal carcinoma), HCT116 (colorectal carcinoma) and hCMEC/D3 (human brain capillary endothelial cells), respectively—using the MTT [3-(4,5-dimethylthiazol-2-yl)-2,5-diphenyltetrazolium bromide] assay, as previously described (Girão et al., 2019). The crude extract was tested in triplicate, in two independent experiments. Results are presented as percentage of cellular viability relative to the solvent control, after 48 h of exposure. Data from cytotoxic assays (six technical replicates in total per sample) was tested for significant differences compared to the solvent control, and the significance level was set for all tests at *p* < 0.05. The normality distribution of data was accessed using the Kolmogorov Smirnov test. One-Way ANOVA was applied followed by Dunnett’s *post hoc* test for parametric data, and Kruskal–Wallis test, followed by Dunn’s multiple comparison test, used for non-parametric data. The apoptotic Staurosporine (Sigma-Aldrich, MO, United States) was used as positive control, at the same concentration as the extracts, and 0.5% DMSO was used as solvent control.

**TABLE 1:** Reference strains used in the antimicrobial assay with the indication of the culture medium used, incubation time and temperature.

Human pathogens	Culture medium	Incubation time (hours)	Incubation temperature (^◦^C)
*Escherichia coli* ATCC25922	Mueller-Hinton	18	37
*Salmonella enterica* ATCC25241	Mueller-Hinton	18	37
*Bacillus subtilis* ATCC6633	Mueller-Hinton	18	37
*Staphylococcus aureus* ATCC29213	Mueller-Hinton	18	37
*Candida albicans* ATCC10231	Sabouraud Dextrose	18	37
Fish pathogens			
*Edwardsiella tarda* DSM30052	Tryptic Soy	24	28
*Aeromonas hydrophila* DSM3018	Tryptic Soy	24	28
*Pseudomonas anguilliseptica* DSM12111	Tryptic Soy	48—72	25
*Yersinia ruckeri* ATCC29473	Tryptic Soy	24—48	30
*Listonella (Vibrio) anguillarum* ATCC19264	Tryptic Soy	48	30
*Tenacibaculum maritimum* ATCC43397	Marine Broth	24—48	26

### Decylprodigiosin isolation and structure elucidation

To obtain sufficient amounts of compounds for a bioactivity-guided isolation, strain CT-F61 was cultivated in a larger scale (24 L) using 5 L Erlenmeyer flasks, each containing 2 L of AIA culture medium. A pre-inoculum of 100 mL was prepared in the same culture medium, using a 250 mL Erlenmeyer flask, to inoculate the bigger flasks. After 7 days of incubation, 30 g of Amberlite XAD16N resin (Sigma-Aldrich, MO, United States) were added to each flask and incubation continued for seven additional days. The biomass and resin were recovered by centrifugation (2500g, 5 min), lyophilized, and repeatedly extracted using a mixture of acetone/methanol 1:1 (v/v). After confirming the previously observed biological activities, by testing the obtained organic extract in the formerly described sets of assays, a reverse-phase vacuum liquid chromatography (VLC) of the organic extract was performed using a solvent polarity gradient (Supplementary Table S1) on a glass chromatography column. All resulting fractions were tested for antimicrobial and cytotoxic activities and examined for the presence of unknown molecules using Global Natural Products Social Molecular Networking (GNPS) dereplication tools (Wang et al., 2016), based on liquid chromatography coupled to high-resolution electrospray ionization mass spectrometry (LC-HRESIMS/MS) analysis. LC-HRESIMS/MS analyses were performed on an UltiMate 3000 UHPLC (Thermo Fisher Scientific) system composed of an LPG-3400SD pump, WPS-3000SL autosampler, and VWD-3100 UV/vis detector coupled to a Q Exactive Focus Hybrid Quadrupole-Orbitrap mass spectrometer controlled by Q Exactive Focus Tune 2.9 and Xcalibur 4.1 (Thermo Fisher Scientific, US). For LC-HRESIMS data, full scan mode was used with the capillary voltage set to −3.8 kV, capillary temperature to 300°C, and sheath gas flow rate to 35 units. The active VLC fractions with no hits for known compounds in the GNPS-based dereplication were selected and further subjected to a reverse-phase semi-preparative high-performance liquid chromatography (HPLC) (flow 3 mL min^–1^, column ACE 10 C18-AR, 250 × 10 mm; Supplementary Table S2). All resulting fractions (Supplementary Figure S1) were tested for bioactivity. Manual dereplication of mass features present in the most active fractions against the Dictionary of Natural Products database (version 32.1—dnp. chemnetbase.com) and the NP Atlas (version 2023_06—npatlas. org) unveiled the presence of an undescribed mass feature *m/z* 380.2699 [M + H]^+^. Fractions containing this mass were pooled and an additional purification step targeting this putative new compound was performed in a reverse-phase analytical HPLC (flow 0.8 mL min^–1^, column ACE Excel 3 Super C18 V19-3214 75 × 4.6 mm; Supplementary Table S3). The chemical structure of this compound was elucidated by comparing its MS/MS data with those of commercial undecylprodigiosin (Wasserman et al., 1966) standard (Abcam, Netherlands). LC-HRESIMS/MS analysis of both molecules was performed by direct injection of each (1.0 mg mL^–1^, flow 0.005 mL min^–1^) into the spectrometer, with a 35,000 fwhm resolution, using an isolation window of 1 m*/z*, loop count of 3, AGC target of 5 × 10^4^, and a collision energy of 35 (arbitrary units). The parent mass of each molecule was selected for fragmentation and the resulting MS/MS spectra fragmentation patterns compared. Additionally, ^1^H (600 MHz) NMR spectroscopy was used to a more comprehensive understanding of the novel compound molecular structure. The NMR data were acquired in methanol-*d*_4_ (CD_3_OD).

### *Streptomyces violaceoruber* CT-F61 genome sequencing

The previously obtained gDNA of strain CT-F61 was sequenced using Illumina 2 × 250 bp paired-end technology. The identification of the closest reference genomes for reading map was performed using Kraken 2 (Wood et al., 2019), reads quality check was done using BWA-MEM and final assembly was established using SPAdes (Bankevich et al., 2012). DFAST (Tanizawa et al., 2018) was used to determine completeness and contamination. The genome sequence was annotated using the NCBI Prokaryotic Genome Annotation Pipeline and deposited at GenBank under the accession number SAMN37527650. AntiSMASH 6.0 (Blin et al., 2021) was used for the automated analysis and identification of secondary metabolite biosynthetic gene clusters using relaxed detection settings and all extra features selected.

## Results and discussion

### *Streptomyces violaceoruber* CT-F61 isolation and taxonomic identification

**FIGURE 1:**
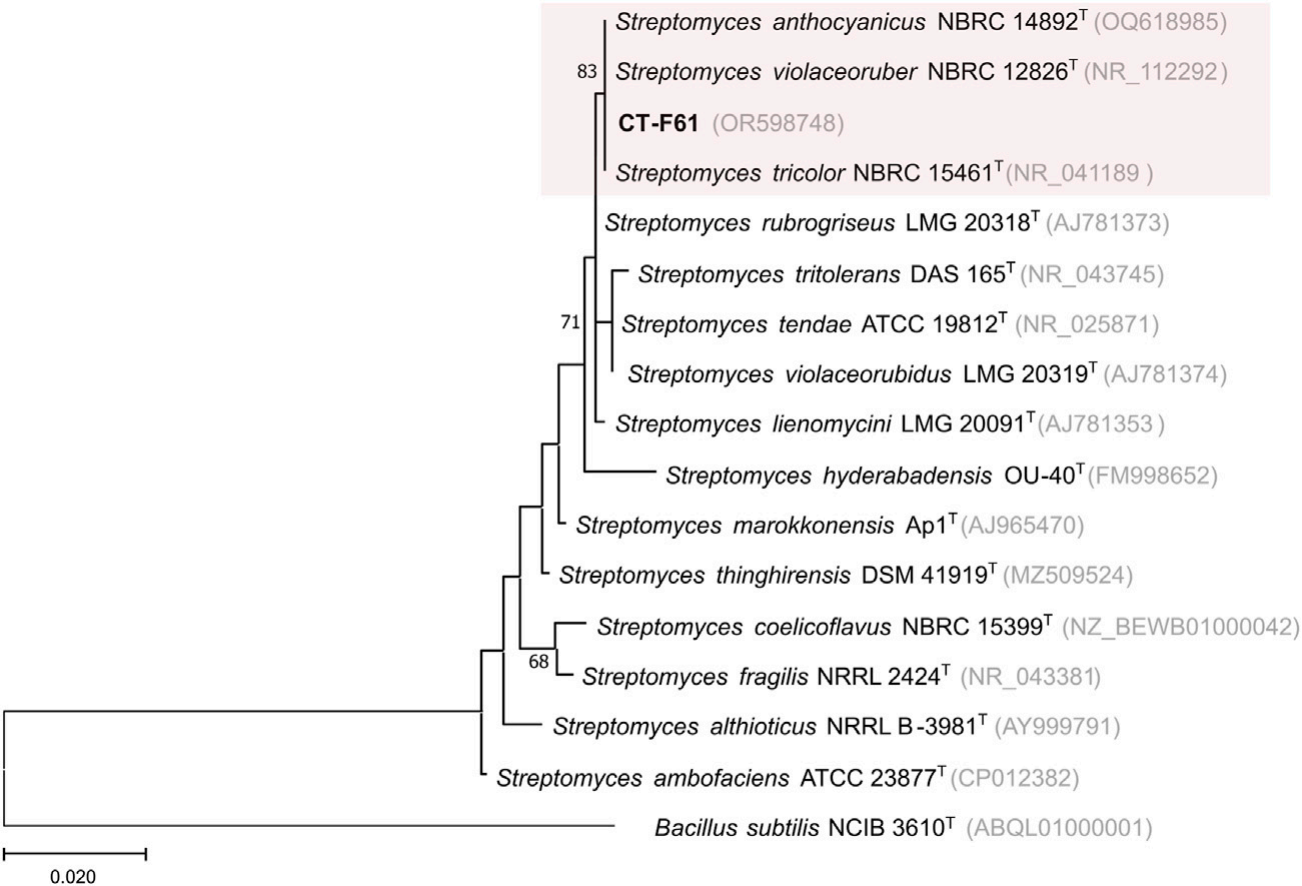
Maximum-likelihood phylogenetic tree, based on 16S rRNA gene sequences (1366 nt), showing the relationship between strain CT-F61 and closest related type species. Accession numbers are indicated in brackets. Values at the nodes indicate bootstrap values higher than 50%, obtained from 1000 resampling events. *Bacillus subtilis* NCIB 3610T was used as outgroup.

Our continued efforts in exploring seaweed-associated actinobacterial diversity led to the isolation of *Streptomyces violaceoruber* CT-F61 from the Chlorophyta *Codium tomentosum*. From the macroalgae frond tissues, using the selective culture medium AIA, formulated with 40% of seawater, a regular, opaque, white spore forming colony, able to change the culture medium colour from yellowish to dark blue, was isolated from the frond tissues of *C. tomentosum*, purified, and named as strain CT-F61. According to the EzBioCloud 16S database, strain CT-F61 showed the highest 16S rRNA gene sequence similarity to *S. violaceoruber* NBRC 12826^T^, *Streptomyces anthocyanicus* NBRC 14892^T^ and *Streptomyces tricolor* NBRC 15461^T^ (all 99.85%), three species that have been recently classified as heterotypic synonyms of *S*. *violaceoruber* based on multilocus sequence analysis (Komaki, 2022). Phylogenetic assessment showed that strain CT-F61 clustered with the three type strains mentioned above (Figure 1). Although 16S rRNA gene is traditionally used in bacterial systematics, its resolution might not be sufficient for species identification, especially for genera integrating a large number of species, like the *Streptomyces* genus. Genome sequencing of strain CT-F61 showed a closest association to *S. anthocyanicus* JCM 5058, with 99.36% average nucleotide identity (ANI) between the two strains, based on Genome Taxonomy Database (GTDB) taxonomy assignment. Considering all the mentioned aspects, strain CT-F61 was identified as *Streptomyces violaceoruber* CT-F61.

### Bioactivity screening

**FIGURE 2:**
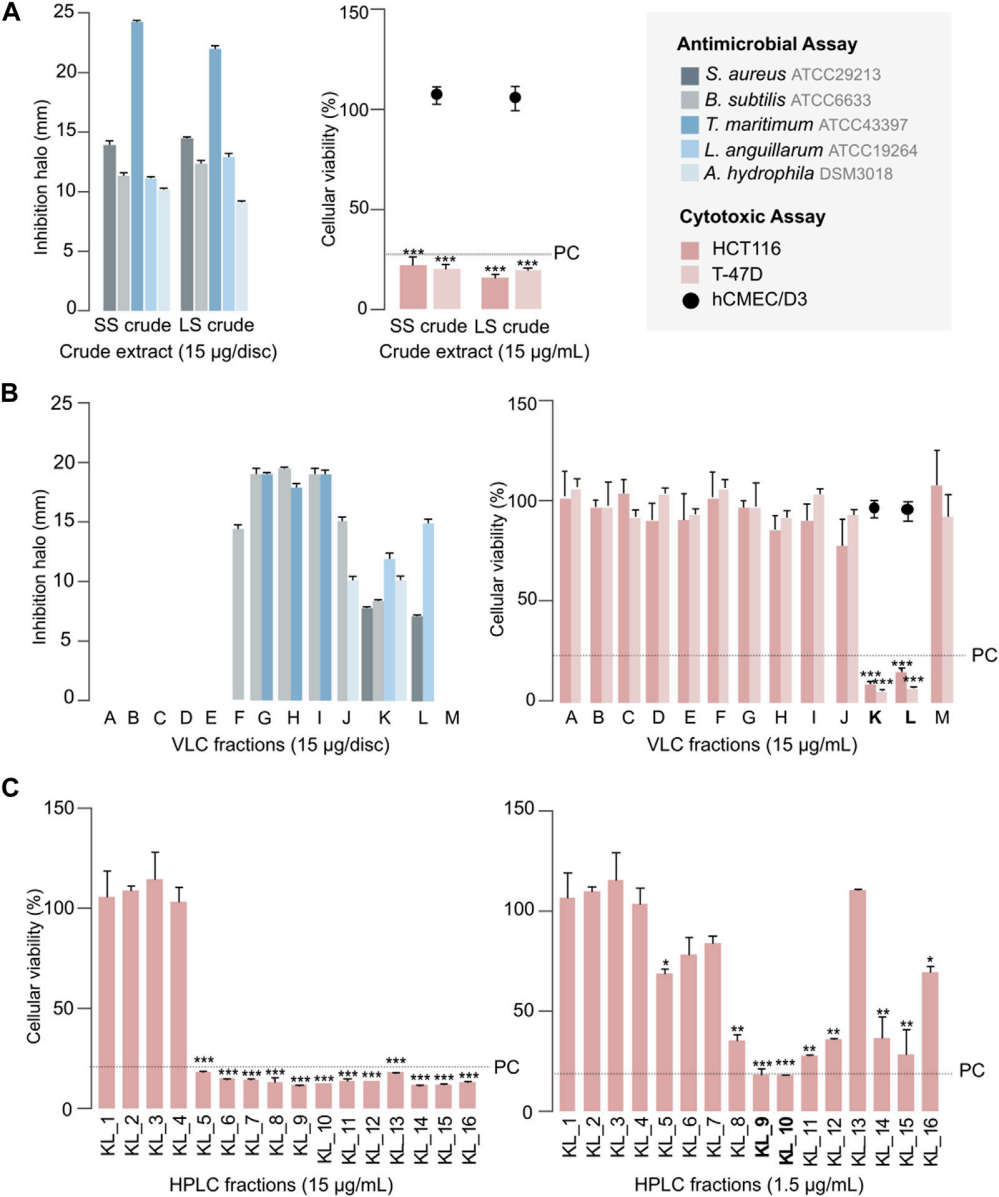
Antimicrobial and cytotoxic activities of CT-F61 crude extract, obtained from the small-scale (SS) and large-scale (LS) cultivation **(A)**, C18 VLC fractions of CT-F61 LS crude extract **(B)** and CT-F61 KL C18 HPLC fractions tested at 15 μg mL^-1^ and 1.5 μg mL^-1^ in HCT116 cell line **(C)**. Antimicrobial results presented as mean of the diameter of the inhibition halos measured from two independent experiments. Only reference strains affected by at least one tested sample are presented. Cytotoxic results presented as percentage of cellular viability after 48 h of exposure, measured as mean from two independent MTT experiments, performed with triplicates to each sample. Significant differences compared to the solvent control (*p < 0.05; **p < 0.01; ***p < 0.001). The percentage of cellular viability for the positive control (PC: Staurporine 15 μg mL^-1^) is indicated, as well as the samples activity on the non-carcinogenic cell line hCMEC/D3.

In order to assess the bioactive properties of *S. violaceoruber* CT-F61, we cultivated this strain in a small scale (SS; 30 mL) with 0.5 g of resin, and tested an organic (acetone/MeOH, 1:1 - both cells and resin material were extracted together) extract from this culture, dissolved in DMSO, against a panel of pathogenic microbial strains and on different human cancer cell lines, using a test concentration of 15 μg mL^-1^. The extract from this seaweed-associated actinomycete inhibited the growth of the Gram-positive bacteria *B*. *subtilis* and *Staphylococcus aureus* (Figure 2A). Exploring new sources of pharmaceutically-relevant compounds is crucial to address major global crisis as antibiotic resistance, responsible for over 700,000 human deaths annually (Church and McKillip, 2021), or the pressing and lacking-solution cancer pathologies (Ferlay et al., 2021). Additionally, CT-F61 proved to inhibit the growth of the Gram-negative fish pathogens *T*. *maritimum, L. anguillarum* and *Aeromonas hydrophila*. *Tenacibaculum maritimum* is a bacterial pathogen responsible for tenacibaculosis, an ulcerative disease causing significant mortalities in various marine fish species worldwide, with high economic impact in aquaculture industry (Mabrok et al., 2023). Similar losses occur due to *L*. *anguillarum,* agent of vibriosis (Hickey and Lee, 2018), and *A. hydrophila* that distresses fishes with gastroenteritis and septicemia (Semwal et al., 2023). Molecules encoded in CT-F61 crude were thus found to have potential to address these diseases, a less-explored biotechnological application of prodigiosins. No inhibitory activity was recorded against the growth of the human pathogens *E. coli* and *Salmonella enterica* and *C. albicans*, and the aquaculture-relevant species *E. tarda*, *P. anguilliseptica* and *Y. ruckeri*. In the cancer cell line assays, the extract reduced the viability of breast carcinoma T-47D and colorectal carcinoma HCT116 cell lines by more than 80%, with no deleterious effect on non-cancer cells (Figure 2A).

### Bioactivity-guided isolation and structure elucidation of decylprodigiosin

**FIGURE 3:**
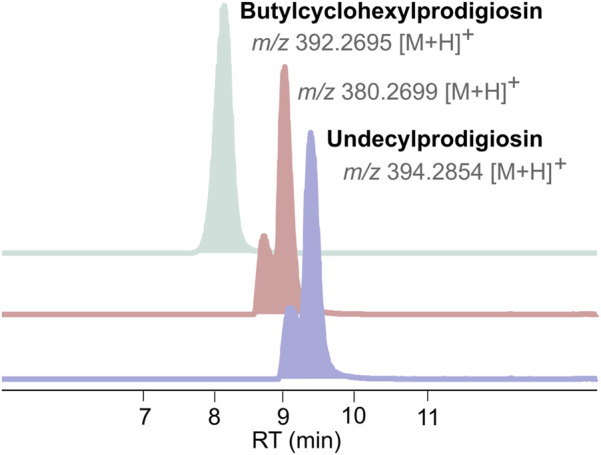
Extracted ion chromatograms (EICs) of Undecylprodigiosin and Butylcyclohexylprodigiosin, detected in KL_9 and KL_10 fractions, alongside with the mass feature corresponding to 1.

**FIGURE 4:**
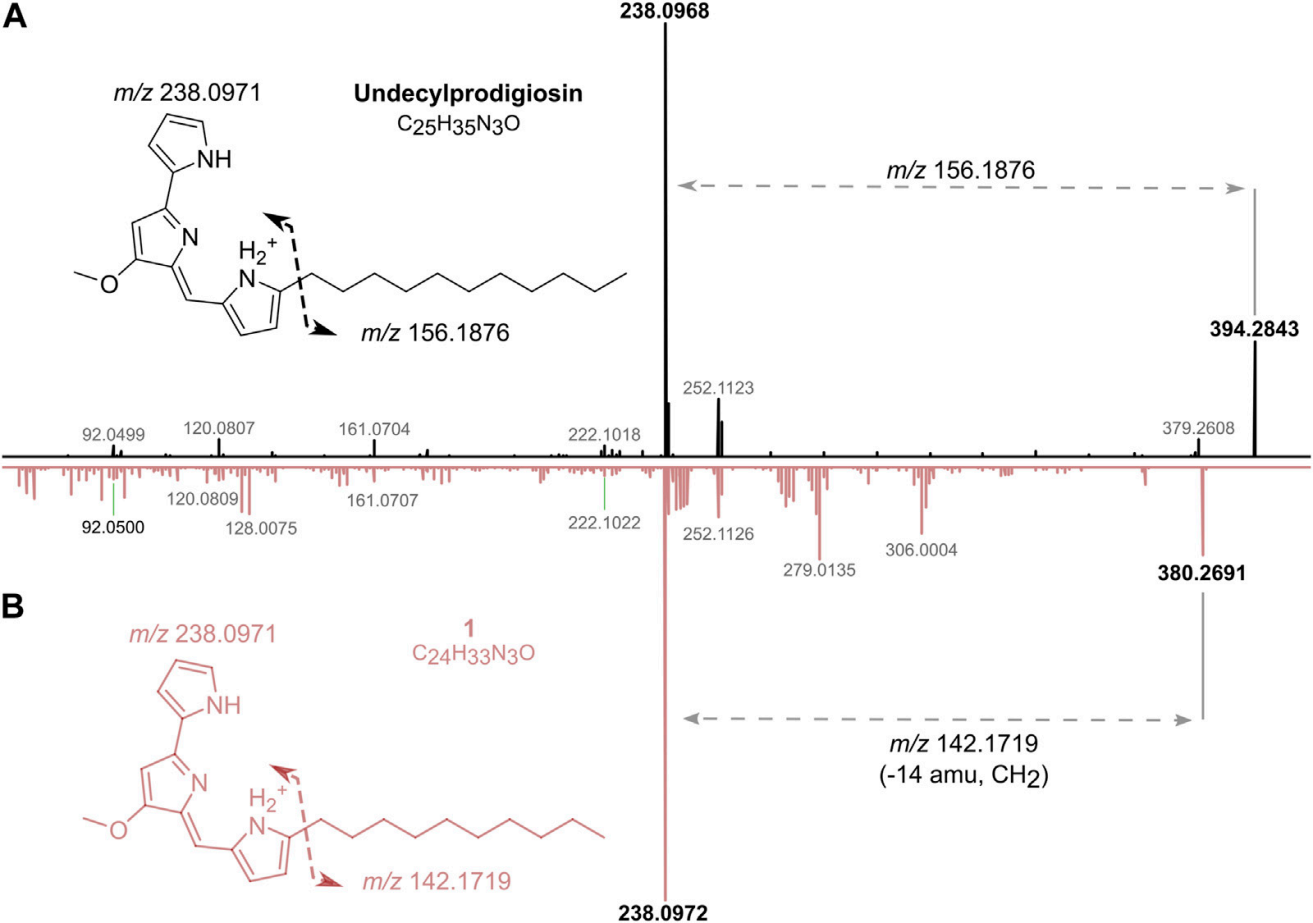
Structure elucidation of one by comparison of HRESIMS/MS spectra of undecylprodigiosin (in black) and one (in pink) **(A)**. Proposed chemical structure of one and molecular formula **(B)**.

Dereplication of the CT-F61 organic extract using GNPS Dereplicator, Dereplicator VarQuest, and Dereplicator^+^ tools (Mohimani et al., 2017; Gurevich et al., 2018; Mohimani et al., 2018) did not lead to the identification of known compounds that could explain the observed biological activity. Thus, we performed large-scale cultivation (LS; 24 L) of the strain, in order to isolate any putative novel bioactive compound from its metabolome. An organic crude extract of 5.7 g, with a similar bioactive profile as the one previously recorded for the SS culture, was obtained (Figure 2A). A set of sequential chromatographic steps was then used to purify the bioactive compounds of interest. All generated fractions were subjected to HRMS dereplication to avoid the isolation of known molecules. The VLC (C18 stationary phase) of the LS crude extract led to 13 fractions (A-M) of decreasing polarity. All fractions were tested for antimicrobial and cytotoxic activities (Figure 2B). Several fractions proved to be effective in inhibiting the growth of the reference bacterial strains, with fraction K being active against all, except *T*. *maritimum.* Fractions K and L presented activity on both cancer cell lines tested (*p* < 0.001), with no effect on the viability of the non-cancer cell line. From all the results recorded, in this work we decided to follow the strong anticancer activity of fractions K and L towards the human cancer cell lines T-47D and HCT116. Fractions K and L were pooled (25.1 mg) and fractionated by C18 semi-preparative HPLC. Sixteen fractions were obtained and tested for cytotoxic activity in HCT116 cell line at 15 μg mL^-1^ and 1.5 μg mL^-1^ (Figure 2C). Fractions KL_5 to KL_16 showed strong cytotoxicity when tested at 15 μg mL^-1^, but at 1.5 μg mL^-1^ only fractions KL_9 and KL_10 retained strong cytotoxic activity. Despite the initial dereplication step, fractions KL_9 and KL_10 were dereplicated using GNPS tools to investigate if the recorded cytotoxic activity was due to any putative novel compound. From this analysis, two mass features associated to undecylprodigiosin and butylcyclohexylprodigiosin, known members of the prodigiosins family, were detected. Yet, a more detailed manual search using the Dictionary of NP database and NP Atlas revealed that the protonated ion [M + H]^+^ at *m/z* 380.2699, detected in the same fractions, was not associated to any described prodigiosin, suggesting that this was a new compound (Figure 3). Is noteworthy to mention that no prodigiosin or related compound was initially detected by GNPS dereplicator tools in the crude extract or VLC fractions, as these were possibly masked by the complex matrix. The presence of undecylprodigiosin and butylcyclohexylprodigiosin could explain the recorded bioactivity, as their anticancer and antibacterial properties are well-recognized. Nonetheless, we decided to focus on the potentially-new prodigiosin analogue. To our knowledge, prodigiosins or related compounds have not been reported so far from strains affiliated to the species *S. violaceoruber*, *S. anthocyanicus* or *S. tricolor* (heterotypic synonyms of *S. violaceoruber).* Fractions KL_9 and KL_10 were combined (6.8 mg) and processed in a C18 analytical HPLC to further purify the new molecule. Based on the NMR data it was clear that the compound contained typical prodigiosin signals, (δ_H_ 7.5–6.20, associated to the pyrrole rings, as well as a large methylene envelope δ_H_ 1.29–1.25), but was not pure. Due to the low amount of compound isolated we decided to approach its structure elucidation using MS/MS. A standard of undecylprodigiosin was acquired and a MS/MS fragmentation comparative study was performed (Figure 4A). Using this approach, we could conclude that **1** m*/z* 380.2699 [M + H]^+^ differs from undecylprodigiosin on the carbon alkyl chain with a loss of a methylene group (−14 atomic mass units), conserving the aromatic moieties (*m/z* 238.0971). Based on this 10-carbon alkyl chain feature and the absence of ^1^H NMR signals pointing towards a terminal isopropyl moiety, this chain is proposed to be linear and the compound was designated as decylprodigiosin (**1**, Figure 4B). Reported isopropyl-containing alkyl chains in prodigiosins are odd-numbered (van Santen et al., 2022). Additional studies must be performed to characterize the bioactive properties of this novel molecule. To confirm that strain CT-F61 contains the genetic information necessary to produce prodigiosins, we sequenced its genome. The genome data was assembled into one contig with a length of 8 599 857 bp, with *in silico* G + C content of 72.2 mol%. DFAST results of completeness and contamination were 99.92% and 0.08%, respectively. As predicted, using AntiSMASH we identified a genomic region in the genome of strain CT-F61 in which the entire set of genes associated with the production of undecylprodigiosin (biosynthetic gene cluster from *Streptomyces coelicolor* A3 (2)—MIBiG accession: BGC0001063) could be found. The even-numbered chain in **one** could be derived from an odd-numbered starter unit extended by the polyketide synthase machinery involved in the biosynthesis of actinobacterial prodigiosins (Hu et al., 2016).

### Prodigiosins as product of seaweed-associated actinobacterial metabolism

The intricate web of symbiotic relationships in nature can shape entire ecosystems. In aquatic environments, symbiosis plays a key role in entire bionetworks, as, for example, in the coral-algae mutualism supporting a quarter of marine life (Tirichine and Piganeau, 2023). Seaweeds offer a suitable substratum for bacterial life and provide organic nutrients for multiplication and establishment of biofilms. In return, the host benefits from chemicals synthetized by the bacterial communities that can act as growth-promoting substances, quorum sensing signaling molecules or bioactive compounds responsible for their normal morphogenesis, growth and survival (Egan et al., 2013; Singh and Reddy, 2014). One distinctive trait of the red-pigmented family of the antibiotics prodigiosins is their algicidal activity (Zhang et al., 2016; Yang et al., 2017; Zhang et al., 2017; Zhang et al., 2020). In this work we show for the first time that a symbiotic *Streptomyces* strain, isolated from the tissues of a green macroalgae, is able to produce a wide range of bioactive prodigiosins. Prodigiosin and its family derivatives have been widely studied due to their biotechnological applications. In particular, this NPs family is efficient across several cancer types with low effects against non-malignant tissues, also offering interesting possibilities for combinatorial applications once they can act synergistically and/or additively with other drugs (Manderville, 2001; Montaner and Prez-Toms, 2003; Anwar et al., 2022). Different prodigiosin analogues, with minor modifications on their structures, have shown different modes of action and degrees of cytotoxicity (Kimata et al., 2018). Therefore, the discovery of a new prodigiosin molecule can provide additional insights into the structure-activity relationships within this natural products family. Even without certainty about the ecological role that these compounds play in this marine niche, we hypothesize that prodigiosins may be involved in protecting the host from algal overgrowth. In this symbiotic relationship, the seaweed provides a hospitable environment for *Streptomyces* to thrive, while the bacterial partner reciprocates by potentially deploying its potent prodigiosins arsenal. Such a natural defense mechanism could prevent excessive algal colonization that otherwise would compete with the seaweed host for vital resources such as sunlight and nutrients. However, more studies should be conducted to test this possibility.

## Conclusion

In this work we have explored the bioactive secondary metabolism of the seaweed-associated *Streptomyces violaceoruber* CT-F61*,* isolated from the tissues of *Codium tomentosum,* a green macroalgae from the northern Portuguese shore. We describe the antimicrobial and anticancer properties of the metabolome of this strain, valuable for both human and animal health. We describe the discovery of 1, a new 10-carbon alkyl chain member of the prodigiosin family. To our knowledge, no prodigiosin or prodigiosin-like molecule has been described before from an actinomycete living is symbiosis with a seaweed, proving the value of this ecological niche as a source of novel NPs with biotechnological applications. Additional studies should be performed to allow a better understanding of the bioactivity and ecological role of 1.

## Data Availability

The datasets presented in this study can be found in online repositories. The names of the repository/repositories and accession number(s) can be found below: https://www.ncbi.nlm.nih.gov/genbank/, SFRH/BD/145646/2019 https://www.ncbi.nlm.nih.gov/genbank/, SAMN37527650 https://massive.ucsd.edu/ProteoSAFe/static/massive.jsp, MSV000093436 https://gnps.ucsd.edu/ProteoSAFe/libraries.jsp, CCMSLIB00012176068.
