# Supplementary material for: Decylprodigiosin: a new member of the prodigiosin family isolated from a seaweed-associated Streptomyces
Source: Front Pharmacol. 2024 Mar 21;15:1347485. doi: 10.3389/fphar.2024.1347485 (PMC10991731; doi:10.3389/fphar.2024.1347485)
Supplement: Supplementary file 1 [file DataSheet1.pdf]

Supplementary Material

Supplementary Figures and Tables

Table S1. Reverse-phase VLC conditions of strain CT-F61 crude extract.

Fraction	MeOH (%)	H_2O (%)	Volume (mL)
A	5	95	500
B	10	90	500
C	20	80	500
D	30	70	500
E	40	60	500
F	50	50	500
G	70	30	500
H	90	10	500
I	100	0	750
J	100 IPA	-	1000
K	100 DCM	-	1000
L	100 AC	-	500
M	100	0	750

MeOH: metanol; H_2O: water: IPA: isopropanol;
DCM: dicloromethane; AC: acetone

Table S2. Reverse-phase semi-preparative HPLC conditions of CT-F61 KL sample.

Time (min)	% H_2O	% MeOH	% IPA
0.00	70	30	0
12.00	0	100	0
25.00	0	30	70
27.00	0	20	80
42.0	0	20	80
44.0	0	100	0
45.0	7	30	0
46.0	70	30	0

MeOH: metanol; H_2O: water; IPA: isopropanol

Supplementary Figure 1. Reverse-phase HPLC chromatogram of CT-F61 KL sample with the indication of the recovered fractions.

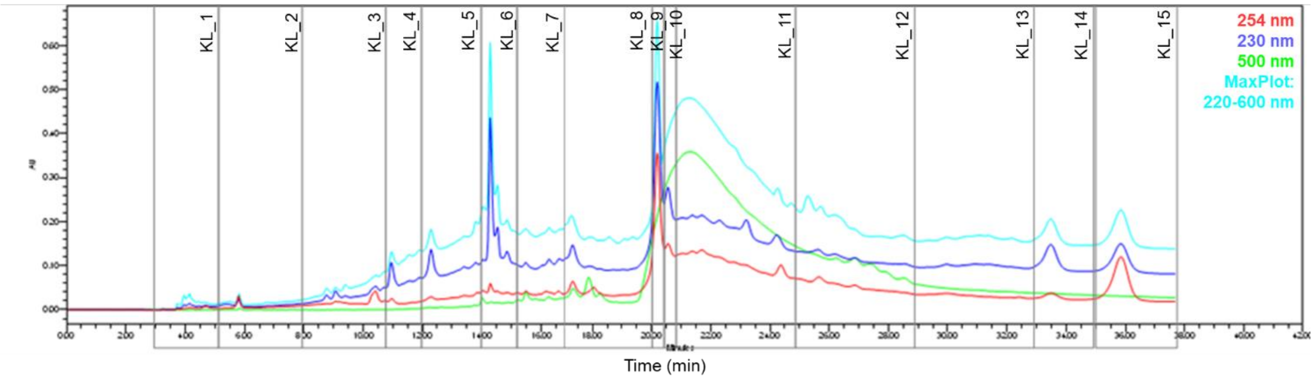

Table S3. Reverse-phase analytical HPLC conditions of CT-F61 KL_9-10 sample. Column used: ACE Excel 3 Super C18 V19-3214 75x4.6 mm.

Time (min)	% H_2O	% MeOH	% IPA
0.01	50	50	0
18.00	0	100	0
23.00	0	70	30
30.00	0	70	30
33.0	50	50	0
37.0	50	50	0

MeOH: metanol; H_2O: water; IPA: isopropanol

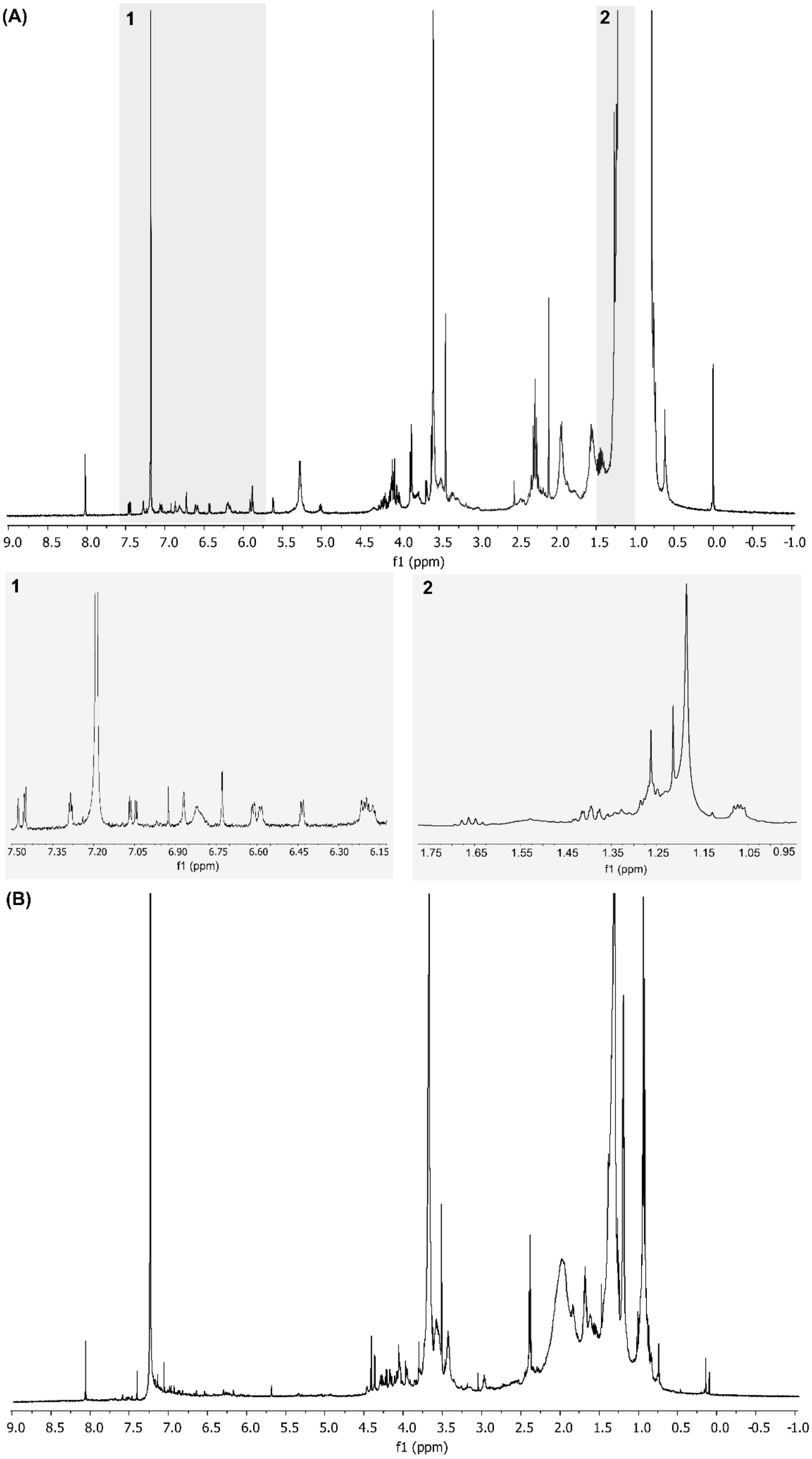
(A)
1
2
9.0 8.5 8.0 7.5 7.0 6.5 6.0 5.5 5.0 4.5 4.0 3.5 3.0 2.5 2.0 1.5 1.0 0.5 0.0 -0.5 -1.0
f1 (ppm)
1
7.50 7.35 7.20 7.05 6.90 6.75 6.60 6.45 6.30 6.15
f1 (ppm)
2
1.75 1.65 1.55 1.45 1.35 1.25 1.15 1.05 0.95
f1 (ppm)
(B)
9.0 8.5 8.0 7.5 7.0 6.5 6.0 5.5 5.0 4.5 4.0 3.5 3.0 2.5 2.0 1.5 1.0 0.5 0.0 -0.5 -1.0
f1 (ppm)

Supplementary Figure 2. ^{1}H NMR (Chloroform-d, 600 MHz) spectrum of fraction KL_9 containing semipurified **1**, highlighting the typical prodigiosin signals δ_H 7.5-6.20, associated to the pyrrole rings **(A)** and δ_H 1.29-1.25, consistent with a large methylene envelope **(B)**. ^{1}H NMR (Chloroform-d, 600 MHz) spectrum of the most purified fraction (KL_9-10_A) containing **1 (C).**
